# Neurofeedback technique for treating male schizophrenia patients with impulsive behavior: a randomized controlled study

**DOI:** 10.3389/fpsyt.2024.1472671

**Published:** 2024-10-07

**Authors:** Zhenkuo Li, Hao Ren, Yinghan Tian, Jiqian Zhou, Wenhao Chen, Guohua OuYang, Zhaolai Chen, Wei Yi, Hongli Song, Peng Xie, Xinchun Wang, Xi Chen, Qinglin Xiao, Huanzhong Liu

**Affiliations:** ^1^ Department of Psychiatry, The Fifth People’s Hospital of Xiangtan City, Xiangtan, China; ^2^ Department of Psychiatry, Chaohu Hospital of Anhui Medical University, Hefei, China; ^3^ Department of Psychiatry, Chongqing Changshou District, Mental Health Center, Chongqing, China

**Keywords:** neurofeedback therapy, male, schizophrenia, agitation, impulsiveness, PANSS-excited component

## Abstract

**Background:**

Schizophrenia is one of the most severe mental disorders, frequently associated with aggression and violence, particularly in male patients. The underlying mechanisms of violent behavior in these patients remain unclear, limiting effective treatment options and highlighting the need for further research into interventions for impulsive behaviors. This study aims to evaluate the clinical efficacy of neurofeedback treatment in hospitalized male schizophrenia patients exhibiting impulsive behaviors.

**Methods:**

The study was designed as a single-center, randomized, single-blind, sham-controlled parallel trial. Eighty patients were randomly assigned to either a study group or a control group. The control group received risperidone and sham neurofeedback, while the study group received risperidone and active neurofeedback therapy. Both groups underwent training five times per week, with each session lasting 20 minutes, over a six-week period. Clinical symptoms were assessed at baseline, three weeks and six weeks using the Positive and Negative Syndrome Scale (PANSS), the Modified Overt Aggression Scale (MOAS), and the Rating Scale for Extrapyramidal Side Effects (RSESE). Statistical analyses were conducted to compare the therapeutic effects between the two groups at the study’s conclusion.

**Results:**

Initial comparisons showed no significant differences in baseline data, except for the number of prior hospitalizations (P<0.018). By the end of the study, the study group demonstrate significant improvements in MOAS and PANSS scores (including the Excited, Positive, Cognitive, and Depressive/Anxiety Components), with no significant changes in RSESE scores.

**Discussion:**

Both time and group interactions were significant across most outcomes, underscoring the efficacy of neurofeedback in reducing the severity of impulsive behaviors and associated schizophrenia symptoms.

**Clinical trial registration:**

chictr.org.cn, identifier ChiCTR2200063407

## 1 Introduction

Schizophrenia, characterized by persistent cognitive, emotional, behavioral, and volitional impairments, is a leading cause of disability worldwide, with a high potential for relapse that results in significant social dysfunction. It is recognized as one of the top ten most disabling global diseases, often resulting in severe adverse outcomes and considerable burden on the families of affected individuals (1, 2). Compared to the general population, impulsivity and violent behaviors are markedly more prevalent in individuals with schizophrenia (3). Longitudinal studies published in *The Lancet Psychiatry* indicate that the incidence of such behaviors in schizophrenia is significantly higher than in other mental disorders over periods of 5 to 10 years (4). Notably, impulsive and violent actions are considerably more prevalent in male patients than in female patients (5–7). These actions often involve recklessness and cruelty and are typically executed in public settings (8).

Impulsive and violent behaviors in male schizophrenia patients have attracted significant social attention due to the substantial economic burden and safety concerns they pose for families and communities. The neuroelectrophysiological and pathological underpinnings of these behaviors remain elusive, leading to a lack of targeted treatments. Modified Electroconvulsive Therapy (MECT) is widely used in clinical settings to address acute schizophrenia symptoms, such as agitation. However, the use of anesthetics and electroconvulsive methods during MECT can impair emotional and cognitive functions and may even alter brain structures (9, 10). The risks associated with anesthesia and electroshock in this treatment process also require careful consideration (11). Consequently, this treatment often elicits anxiety and resistance among patients and their families. Although antipsychotic drugs and mood stabilizers are effective in reducing impulsive and agitated behaviors (12), the potential side effects, including Neuroleptic Malignant Syndrome (NMS) (13), QT prolongation syndrome, sudden death, and respiratory suppression, are significant concerns (14).

In recent years, neurofeedback therapy has gained prominence for its non-invasive nature and minimal side effects, showing promise in treating various mental disorders (15). It has been applied to alleviate symptoms in conditions such as autism (16), depression (17), and schizophrenia, particularly its negative symptoms (18). This therapy modulates brainwave frequencies by enhancing or suppressing specific waves to achieve desired therapeutic outcomes. Typically, alpha waves (8-13Hz), which are dominant in magnetoencephalograms of healthy individuals’ (19), are targeted. These waves, generated through cortical interactions influenced by thalamic rhythms, diminish in individuals displaying increased excitability and impulsive or violent behaviors, suggesting a reduced cortical inhibitory function (20). Studies have consistently shown that reduced alpha wave activity in the temporal and occipitoparietal regions correlates with violent behaviors in males with mental disorders (21). Furthermore, reductions in alpha rhythms have been identified as a key electrophysiological change in violent offenders with antisocial personality disorder (22). Additionally, several studies indicate that schizophrenia patients exhibit lower alpha wave amplitudes, with a notable negative correlation between alpha amplitude and the severity of psychotic symptoms, particularly impulsivity and violent behaviors (23–27). This background provides a theoretical foundation and a practical approach for the current study, which involves alpha wave enhancement training in male schizophrenia patients with impulsive behaviors. The aim is to increase cortical inhibitory capacity, thereby reducing the occurrence of impulsive behaviors. This study focuses on neurofeedback training that enhances alpha waves over a six-week period, with sessions conducted five times per week, to diminish impulsive behaviors and improve clinical symptoms through the modulation of neuroelectrophysiological signals.

Our study introduces a novel, non-invasive treatment modality for male schizophrenia patients exhibiting impulsive behaviors, broadening the range of clinical interventions available for managing impulsivity in mental disorders. This advancement is particularly crucial for increasing clinical effectiveness in these patients by significantly reducing occurrences of impulsive violence and potentially decreasing the incidence of violent criminal behavior among this group.

## 2 Materials and methods

### 2.1 Study design and participants

The study enrolled 80 male inpatients with schizophrenia exhibiting impulsive behaviors, recruited from Xiangtan City’s Fifth People’s Hospital between September 1, 2022, and August 31, 2023. Participants met the following inclusion and exclusion criteria:

Inclusion Criteria:

1. Diagnosed with schizophrenia according to the International Classification of Diseases-10.
2. Male, aged 18-65 years, with no significant physical illnesses, and able to participate in neurofeedback therapy.
3. Minimum one-month prior treatment with standardized oral risperidone (3-6 mg/day), without concurrent therapies (physical, psychological). Currently they should be in a stable (non-acute) phase, have never been treated with MECT and mood stabilizers (lithium, valproate, etc.) or sedative-hypnotics (benzodiazepines, barbiturates, etc.) in the last month.
4. Documented history of impulsive behavior.
5. Scores of ≥60 on the Positive and Negative Syndrome Scale five-factor model (PANSS-5F) and ≥14 on the PANSS-Excited Component (PANSS-EC), with a score of ≥4 on at least one of the five items (28, 29).

Exclusion Criteria:

1. Contraindications to neurofeedback therapy, including a history of epilepsy, severe physical illnesses, or other organic brain diseases.
2. Concurrent substance abuse.
3. Co-existing intellectual disabilities.
4. Persistent impulsive behavior that precludes cooperation with the treatment.

**Figure 1:**
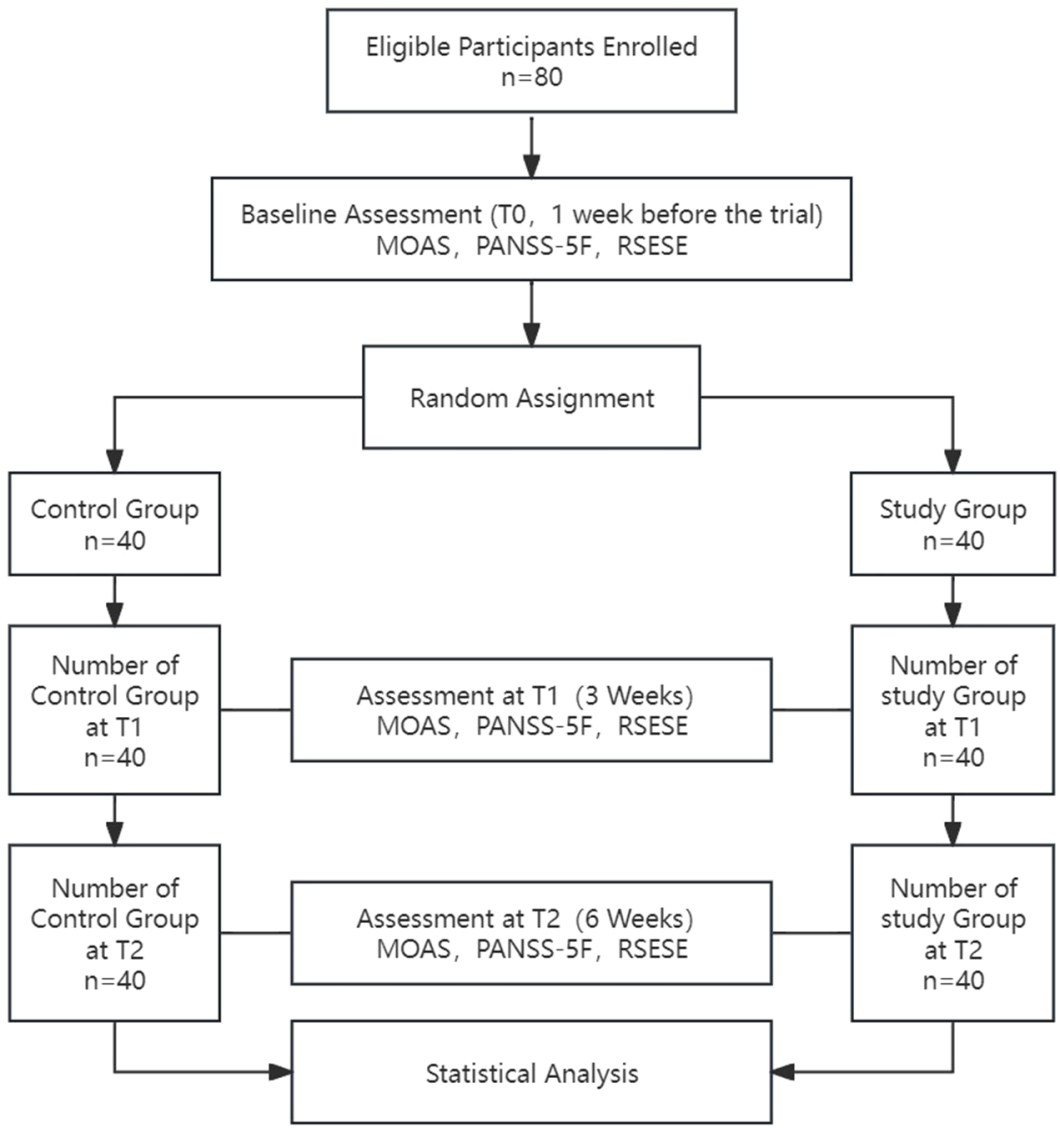
Flow diagram of the study.

This study was approved by the Ethics Committee of the Fifth People’s Hospital of Xiangtan City (approval number: 2022003) and was conducted in full compliance with the Declaration of Helsinki, with no conflicts of interest. All participants were provided with detailed information about the study and voluntarily signed informed consent forms, ensuring their cooperation and the protection of their privacy rights throughout the research process. All staff involved in the study, including psychiatrists, psychiatric nurses, and rehabilitation physicians administering neurofeedback therapy, completed standardized training before the study began. These rehabilitation trainers involved in the treatment all hold certificates of accreditation issued by the relevant administrative departments in China, and can legally perform tasks related to EEG systems. The progression of the study is illustrated in **Figure 1**.

### 2.2 Randomization and blinding

The study was designed as a single-center, randomized, single-blind, sham-controlled parallel trial. Patients were randomly allocated into two groups using a random number table: a study group (n=40) and a control group (n=40). Blinding was implemented according to a neurofeedback treatment protocol (30), ensuring that all participants were unaware of their group assignments and the authenticity of the neurofeedback they received was authentic. Due to the nature of the intervention, rehabilitation therapists administering the feedback were necessarily aware of whether the feedback was real or sham but maintained strict confidentiality throughout the treatment process. Both groups received identical pre-training guidance from the feedback system. However, during the sessions, the neurofeedback device for the control group was offline. Therapists manually operated the system to display random game animations unrelated to actual feedback signals until the completion of the training. Blinding was maintained until after the final data collection.

### 2.3 Intervention

#### 2.3.1 Trial equipment

For group neurofeedback therapy sessions, the BBB-2A device from Guangzhou Runcie Medical Instruments Co., Ltd., China, was utilized. This device used in our study is not approved by the FDA. However, it has been approved for clinical use by the National Medical Products Administration (NMPA) in China. This system allowed therapists to control sessions from a central terminal, enabling simultaneous treatment of up to 20 patients. Continuous EEG raw signals were captured using the device’s integrated 3-channel EEG cap, adhering to the international 10-20 system placement at FP1, FP2, and FPz. FP2 was used as the reference electrode, while FP1 served as the ground electrode. To ensure signal quality, the impedance at all electrode sites was kept below 5 kΩ. The system operated at a sampling rate of 1024 Hz, with a band-pass filter range of 1-30Hz and a notch filter at 50 Hz to eliminate electrical interference.

#### 2.3.2 Neurofeedback therapy protocol

The study involved two groups of participants: the control group received risperidone coupled with sham neurofeedback treatment, while the study group received risperidone alongside actual neurofeedback treatment. Treatment sessions were conducted five times a week, lasting 20 minutes each, over a six-week period (31). Participants were excluded from the study if alterations in medication or dosage were necessary due to changes in psychiatric symptoms. We refer to *Evidence-Based Practice on Biofeedback and Neurofeedback*, edited by Prof. Surmeli (57), to set up the research group’s training regimen: Therapists tailored the neurofeedback training by selecting from various animated. Before starting the treatment, participants followed guided relaxation instructions provided by the software. Once the relaxation was finished, the EEG cap collected up to 10 seconds of electroencephalogram power spectral data. The feedback instrument analyzed this data to calculate the power ratio of alpha waves across all frequency bands from 1-35 Hz. At the same time, the instrument automatically set the feedback threshold according to the power ratio. The bonus percentage was automatically set based on the difficulty level of the game in the feedback treatment, the higher the difficulty the lower the percentage. During the feedback training, patients were required to watch a game animation on the computer screen with their eyes open throughout the session. They were guided by the background music and cues from the game to maintain their EEG alpha wave activity above a certain threshold to keep the animation playing until the end of the training. Instructions within the module aided patients in completing the neurofeedback tasks, encouraging them to fully immerse in and mentally engage with the experience. At the end of each session, four rehabilitation trainers were assigned to discuss the treatment with the patients and complete the feedback sheets. Since the study involved group neurofeedback sessions, with up to 20 patients receiving treatment simultaneously, each trainer communicated with about five patients and completed their treatment documentation. As the sessions progressed, therapists adjusted the difficulty of the training based on the patient’s performance to continuously tailor the therapeutic approach. Participants were also withdrawn if they consistently failed to complete the designated training tasks during the neurofeedback therapy.

### 2.4 Outcome measures

To evaluate the clinical psychiatric symptoms of the patients, the study employs the PANSS-5F scale. The severity of aggressive behaviors was measured using the Modified Overt Aggression Scale (MOAS), while side effects were assessed with the Rating Scale for Extrapyramidal Side Effects (RSESE). Additionally, the study tracked the number of seclusion and mechanical restraint incidents to gauge episodes of impulsivity or agitation (32). These incidents were defined as the total occurrences from the beginning of the study to its conclusion, with each episode involving agitation or impulsivity counted as a single incident, from initiation to release.

Researchers gathered both demographic information (such as age, ethnicity, and marital status) and general clinical data (such as age at onset and number of hospital admissions) for both patient groups at baseline using a specifically designed scale. The PANSS-5F, MOAS, and RSESE were utilized to assess all participants at three pivotal times: before the intervention (T0), at the third week (T1), and at the end of the sixth week (T2). Additionally, we collected root mean square (RMS) value of the alpha wave for the study group both at baseline and at the conclusion of the sixth week. Unfortunately, due to the control group’s host being offline during the sham neurofeedback stimulation, we were unable to gather RMS value of the alpha wave for this group, leading to missing data in this segment.

#### 2.4.1 Primary outcomes

In the study, agitation and impulsivity were evaluated using the PANSS-EC and the MOAS, which were designated as the primary outcomes. The PANSS-EC, a subscale of the PANSS-5F, is widely utilized to measure agitation or impulsivity in clinical pharmacotherapy trials (33, 34).It includes five specific items—hostility, uncooperativeness, impulsivity, tension, and excitability—each rated on a scale from 1 (nonexistent) to 7 (extreme). A higher score on the PANSS-EC reflects greater severity of agitation or impulsivity. The MOAS, on the other hand, assesses the most severe forms of aggression displayed by a patient over the previous month and includes categories such as verbal aggression, physical aggression towards others or oneself, and aggression towards objects (35, 36). The total score on the MOAS indicates the overall severity of aggressive behaviors.

#### 2.4.2 Secondary outcomes

The study designated the scores on the PANSS-5F, PANSS Negative Component (PANSS-Ne), PANSS Cognitive Component (PANSS-Co), PANSS Depressive/Anxiety Component (PANSS-DA), PANSS Positive Component (PANSS-Po), RMS value, and RSESE, as well as the number of protective restraint events, as secondary outcomes. The PANSS-5F score reflects the overall severity of psychiatric symptoms, with higher scores indicating more severe conditions. Similarly, elevated scores on the PANSS-Ne, PANSS-Co, PANSS-DA, and PANSS-Po suggest increased severity in negative symptoms, cognitive impairments, anxiety/depression, and positive symptoms, respectively. The PANSS-5F model has been validated as an effective tool for capturing multidimensional changes in conditions of schizophrenia patients with impulsivity (37–39). RMS value was employed to evaluate the changes in alpha wave activity in the study group before (T0) and after (T2) the treatment. The RSESE measured the severity of extrapyramidal side effects, with rising scores denoting more severe effects. Monitoring changes in RSESE scores helps evaluate the impact of the study interventions on extrapyramidal functions. The frequency of protective restraint events, a common measure in psychiatric settings, served as an indirect indicator of the distribution and potential reduction of impulsive behaviors among participants; more frequent events imply higher levels of impulsivity.

### 2.5 Statistical analysis

In a study employing neurofeedback treatment, a Repeated Measures Analysis of Variance was used to assess the MOAS, reporting an effect size of 0.36 (40). Based on this effect size, with a significance level (α) set at 0.01 and a statistical power (1-β) of 0.8, the necessary total sample size was calculated to be 64 using G-Power 3.1.9.2 (41).

After the 6-week treatment period, data were collated and collected for analysis using IBM SPSS Statistics 25.0. All participants who received at least one instance of the randomized treatment and had one set of baseline data collected were included in the analysis, adhering to the intention-to-treat (ITT) principle. Continuous variables were analyzed as mean ± standard deviation (Mean ± SD) if they followed a normal distribution, or as median and interquartile range [Mdn (P25, P75)] if not. Categorical variables were presented as frequencies (%). Group comparisons were performed using the Chi-square test, independent samples t-test, or Mann-Whitney U test, depending on the data type.

For repeated continuous measures, a two-way Repeated-Measures ANOVA was employed to evaluate the effects of time (baseline, end of week 3, end of week 6), group, and their interaction on both primary and secondary outcomes. Statistical significance was set at p < 0.05 (two-tailed). In cases where participants did not complete the study, the Last Observation Carried Forward (LOCF) method was used to address missing data, thereby minimizing its impact on the study results.

## 3 Results

### 3.1 Comparison of baseline data between control and study groups

The study enrolled 80 eligible patients with an average age of 43.11 ± 9.18 years and an average illness duration of 85.71 ± 44.01 months. The flowchart of the study protocol is depicted in **Figure 1**. Baseline comparisons between the control and study groups revealed a statistically significant difference only in the number of hospitalizations (P=0.018). Other baseline data showed no significant differences, as outlined in **Table 1**. The initial alpha wave RMS value for the study group was recorded at 6.78 ± 1.46.

**Table 1:** Comparison of sociodemographic data and clinical characteristics at baseline between the control group and the study group.

	Total (n=80)	Control Group (n=40)	Study Group (n=40)	Statistics	
Variables				χ^2/^t/Z	*P*
Age (year)	43.11 ± 9.18	42.58 ± 8.99	43.65 ± 9.45	-0.521	0.604
Education (year)	9.05 ± 1.96	9.10 ± 1.99	9.00 ± 1.95	0.227	0.821
Marital status				3.275	0.195
Single	55(68.8%)	27(67.5%)	28(70.0%)		
Married	12(15.0%)	4(10.0%)	8(20.0%)		
Divorced	13(16.3%)	9(22.5%)	4(10.0%)		
Employed Status	60(75.0%)	33 (82.5%)	27(67.5%)	2.4	0.121
Household Registration				0.201	0.654
Local Resident	42(52.5%)	22(55.0%)	20(50.0%)		
Non-local Resident	38(47.5%)	18(45.0%)	20(50.0%)		
Positive family history of schizophrenia	6(7.5%)	2(5.0%)	4(10.0%)	0.721	0.396
Age of Onset (years)	31.23 ± 5.06	30.78 ± 5.09	31.68 ± 5.05	-0.794	0.43
Duration of Illness (months)	85.71 ± 44.01	86.73 ± 45.42	84.70 ± 43.10	0.205	0.838
Number of Hospitalizations	4.86 ± 1.57	5.28 ± 1.52	4.45 ± 1.54	2.416	0.018
MOAS	10.09 ± 2.17	9.90 ± 1.97	10.28 ± 2.36	-0.77	0.443
PANSS-5F	83.21 ± 4.01	83.83 ± 4.43	82.60 ± 3.50	1.373	0.174
PANSS-EC	16.90 ± 1.64	17.10 ± 1.63	16.70 ± 1.65	1.090	0.279
PANSS-Po	19.01 ± 2.33	19.45 ± 2.49	18.58 ± 2.09	1.703	0.092
PANSS-Ne	25.89 ± 2.60	25.73 ± 2.76	26.05 ± 2.45	-0.558	0.579
PANSS-Co	13.80 ± 1.27	13.93 ± 1.40	13.68 ± 1.12	0.881	0.381
PANSS-DA	7.61 ± 1.09	7.63 ± 1.25	7.60 ± 0.90	0.102	0.919
RSESE	11.40 ± 1.78	11.25 ± 1.89	11.58 ± 1.92	-0.763	0.448

PANSS, Positive and Negative Syndrome Scale; MOAS, Modified Overt Aggression Scale; PANSS five-factor, Positive and Negative Syndrome Scale five-factor; PANSS-EC, Positive and Negative Syndrome Scale-Excited Component; PANSS-Po, Positive and Negative Syndrome Scale-Positive Component; PANSS-Ne, Positive and Negative Syndrome Scale-Negative Component; PANSS-Co, Positive and Negative Syndrome Scale-Cognitive Component; PANSS-DA, Positive and Negative Syndrome Scale-Depressive/Anxiety Component; RSESE, Rating Scale for Extrapyramidal Side Effects.

### 3.2 Comparison of outcomes between control and study groups after 3 and 6 weeks of intervention

At the conclusion of the study, the control group recorded six instances of protective restraint events, including four cases of aggression towards others and two cases of destructive escape behaviors. In contrast, the study group had two cases of aggression towards others. The difference in the number of protective restraint events between the two groups was not statistically significant (χ² = 2.22, P = 0.136). At time T2, the alpha wave RMS value for the study group was recorded at 8.60 ± 1.84. Compared to the baseline measurement at time T0, the paired sample t-test showed a statistically significant difference (t = -6.59, p < 0.001).

In the repeated measures ANOVA, significant effects were observed across several measures: the Modified Overt Aggression Scale (MOAS) showed significant change with an F-value of 12.21 (P < 0.001, η² = 0.14); the PANSS-5F demonstrated a substantial effect (F = 128.52, P < 0.001, η² = 0.62); the PANSS-EC indicated significant differences (F = 108.30, P < 0.001, η² = 0.58); the PANSS-Po (F = 58.99, P < 0.001, η² = 0.43); the PANSS-Ne (F = 115.18, P < 0.001, η² = 0.60); the PANSS-Co (F = 35.67, P < 0.001, η² = 0.31); and the PANSS-DA (F = 25.49, P < 0.001, η² = 0.25) revealed substantial effects over time. However, no significant effects were found for the RSESE (F = 0.16, P = 0.745, η² = 0.00).

Statistically significant group differences were observed in several assessments within the study. The MOAS reported an F-value of 5.42 (P=0.023, η²=0.07), the PANSS-5F an F-value of 19.61 (P<0.001, η²=0.20), the PANSS-EC an F-value of 12.29 (P=0.001, η²=0.14), the PANSS-Po an F-value of 15.28 (P<0.001, η²=0.16), and the PANSS-Co an F-value of 5.23 (P=0.025, η²=0.06). However, no significant effects were noted for the RSESE (F=0.09, P=0.769, η²=0.00), the PANSS-Ne (F=2.73, P=0.102, η²=0.03), and the PANSS-DA (F=1.36, P=0.247, η²=0.02).

Additionally, significant interaction effects for Time*Group were observed across all primary outcomes: MOAS (F=5.42, P=0.023, η²=0.07), PANSS-5F (F=23.65, P<0.001, η²=0.23), PANSS-EC (F=15.93, P<0.001, η²=0.17), PANSS-Po (F=12.33, P<0.001, η²=0.14), PANSS-Ne (F=20.12, P<0.001, η²=0.21), PANSS-Co (F=10.38, P<0.001, η²=0.12), and PANSS-DA (F=8.07, P=0.001, η²=0.09). The only exception was RSESE, which showed no significant interaction effect (F=2.31, P=0.126, η²=0.03). Comprehensive details of these analyses are provided in **Table 2**.

**Table 2:** Comparison of outcomes between the control group and the study group after 3 weeks and 6 weeks of intervention.

Outcome measures	Control Group mean ± SD	Study Group mean ± SD	Time (F, *P*-value, η^2^)	Group (F, *P*-value, η^2^)	Time*Group (F, *P*-value, η^2^)
MOAS					
T1-T0	-0.03 ± 0.41*	-1.13 ± 0.40			
T2-T0	-0.08 ± 0.49*	-2.53 ± 0.47	12.21, 0.000, 0.14	5.42, 0.023, 0.07	10.83, 0.001, 0.12
PANSS-5F					
T1-T0	-3.60 ± 0.79	-6.25 ± 0.79			
T2-T0	-6.40 ± 1.27	-15.58 ± 1.27	128.52, 0.000, 0.62	19.61, 0.000, 0.20	23.65, 0.000, 0.23
PANSS-EC					
T1-T0	-1.15 ± 0.25	-1.38 ± 0.22			
T2-T0	-1.85 ± 0.34	-3.85 ± 0.34	108.30, 0.000, 0.58	12.29, 0.001, 0.14	15.93, 0.000, 0.17
PANSS-Po					
T1-T0	-0.43 ± 0.29*	-1.35 ± 0.30			
T2-T0	-1.38 ± 0.43	-3.73 ± 0.43	58.99, 0.000, 0.43	15.28, 0.000, 0.16	12.33, 0.000, 0.14
PANSS-Ne					
T1-T0	-1.25 ± 0.25	-2.15 ± 0.25			
T2-T0	-2.20 ± 0.44	-5.23 ± 0.44	115.18, 0.000, 0.60	2.73, 0.102, 0.03	20.12, 0.000, 0.21
PANSS-Co					
T1-T0	-0.55 ± 0.18	-0.58 ± 0.18			
T2-T0	-0.60 ± 0.22	-1.68 ± 0.22	35.67, 0.000, 0.31	5.23, 0.025, 0.06	10.38, 0.000, 0.12
PANSS-DA					
T1-T0	-0.20 ± 0.14*	-0.15 ± 0.14*			
T2-T0	-0.38 ± 0.17*	-1.10 ± 0.17	25.49, 0.000, 0.25	1.36, 0.247, 0.02	8.07, 0.001, 0.09
RSESE					
T1-T0	0.10 ± 0.07*	-0.13 ± 0.07*			
T2-T0	0.15 ± 0.13*	-0.25 ± 0.17*	0.16, 0.745, 0.00	0.09, 0.769, 0.00	2.31, 0.126, 0.03

*: P >0.05; PANSS, Positive and Negative Syndrome Scale; MOAS, Modified Overt Aggression Scale; PANSS five-factor, Positive and Negative Syndrome Scale five-factor; PANSS-EC, Positive and Negative Syndrome Scale-Excited Component; PANSS-Po, Positive and Negative Syndrome Scale-Positive Component; PANSS-Ne, Positive and Negative Syndrome Scale-Negative Component; PANSS-Co, Positive and Negative Syndrome Scale-Cognitive Component; PANSS-DA, Positive and Negative Syndrome Scale-Depressive/Anxiety Component; RSESE, Rating Scale for Extrapyramidal Side Effects.

## 4 Discussion

The primary goal of this study was to investigate the impact of neurofeedback training on impulsivity in male patients with schizophrenia. The results validated the experimental hypothesis, demonstrating significant therapeutic benefits. After six weeks of neurofeedback therapy, the study group exhibited a substantial reduction in scores on the PANSS-EC and the MOAS compared to the control group, confirming the effectiveness of neurofeedback in mitigating impulsive behaviors. Moreover, the study also highlighted broader clinical improvements: there were significantly greater reductions in the PANSS-5F, PANSS-Ne, PANSS-Co, PANSS-DA, and PANSS-Po scores in the study group relative to the control group. These findings suggest that neurofeedback therapy not only reduces impulsivity but also positively affects positive symptoms, negative symptoms, cognitive impairments, and anxiety/depression in schizophrenia patients.

Numerous studies highlight that mental disorders frequently co-occur with neurophysiological dysregulation, illustrating a clear correlation between neurophysiological changes and mental health issues (42–44). Currently, targeting these neurophysiological signals for treatment is a prominent area of research in mental health care, with neurofeedback therapy at the forefront (45). This therapy employs operant conditioning principles, using biofeedback devices to observe various brain waves and selectively train specific neurophysiological wave frequencies to be enhanced or suppressed. Such targeted training aims to ameliorate medical conditions (46). Extensive clinical research supports the use of neurofeedback therapy as a hopeful intervention for patients with mental disorders, including schizophrenia, where disruptions in neurophysiological signals are evident (15, 47–50). Notably, schizophrenia patients who display impulsive behaviors often exhibit slower alpha wave frequency, further substantiating the link between specific neurophysiological patterns and behavioral symptoms (21, 23, 26, 27). In this study, neurofeedback therapy was employed to enhance alpha wave frequency in schizophrenia patients, specifically targeting the reduction of impulsive behaviors and overall psychiatric symptoms. Notable improvements were observed in the study group, with significant reductions in scores on the PANSS-EC and the MOAS during the treatment intervals (T1-T0 and T2-T0). This suggests that the therapy effectively controlled impulsive behaviors, supporting the hypothesis that enhancing alpha wave frequency positively impacts such behaviors in schizophrenia patients (27). Comparable findings were reported by Konicar’ research team, who utilized neurofeedback technology to increase alpha wave frequency in violent criminals with mental disorders. Their results extended beyond psychiatric improvements, demonstrating enhancements in cognitive functions as well (22). Furthermore, the relationship between impulsive violent behavior and reduced alpha wave activity is not restricted to adults. A review highlighted that similar neurophysiological patterns are associated with antisocial and violent behaviors in youths diagnosed with conduct disorders. It indicated that neurofeedback training might also serve as an effective intervention for treating severe conduct disorders in children (51). Throughout the treatment phases T1-T0 and T2-T0, the reduction in PANSS-Po scores was significantly greater in the study group than in the control group, with a statistically significant Time*Group interaction noted. This reduction indicates a decrease in positive symptoms of schizophrenia, which are often linked to aggression in hospitalized patients; Nolan et al. noted that approximately 20% of such aggression is directly related to positive symptoms (52). The observed trend towards a reduction in positive symptoms in our study could also contribute to diminished impulsive behavior. Additionally, the end of the study saw a notable decrease in PANSS-Ne scores in the study group, suggesting an alleviation of negative symptoms. This finding is consistent with the work of Renata and colleagues, who demonstrated significant clinical efficacy using neurofeedback for alpha wave enhancement in schizophrenia patients, particularly those with predominant negative symptoms (18). Moreover, significant improvements were noted in the anxiety and depression scores in our study group. This aligns with findings from studies on biofeedback therapy targeting anxiety and depressive disorders (17, 53). However, the distinctive affective blunting seen in schizophrenia complicates the interpretation of improvements in emotional symptoms. The reduction in anxiety and depression may be attributed to a synergistic effect from enhancements in other PANSS factors, though the specific mechanisms by which alpha wave enhancement influences emotional symptoms in schizophrenia remain unclear.

The study group exhibited significantly greater improvements in PANSS-Co scores throughout the treatment, suggesting enhanced cognitive functions by its conclusion. Surmeli’s team, starting in 2012, implemented a qEEG-guided neurofeedback regimen for patients with schizophrenia. Their study found that this intervention not only reduced psychiatric symptoms but also improved patients’ perceptions of their medication (54). This aligns with findings from a randomized controlled study by Hsueh et al. (55), where healthy individuals undergoing alpha wave enhancement in neurofeedback therapy demonstrated improvements in working and episodic memory. These parallel results underscore the potential of alpha wave enhancement to boost cognitive function in both mental health patients and healthy individuals. However, our study was limited by the absence of specialized psychiatric scales for cognition, preventing a detailed analysis of the relationship between cognitive functions and impulsivity across multiple dimensions. In terms of safety, despite more frequent protective restraint events in the control group, the difference was not statistically significant, indicating that the data from these events alone could not conclusively determine the therapeutic effects between the groups. Furthermore, no significant differences in the RSESE scores were observed in terms of Time, Group, or Time*Group interactions by the study’s end. This suggests that neurofeedback therapy does not elevate the risk of extrapyramidal side effects, affirming its safety as a treatment option.

The study identified that only the number of hospitalizations differed statistically between the study and control groups among the independent variables. No significant differences were observed in other independent variables, including all psychiatric scales, suggesting that this discrepancy did not impact the balance between the groups. By the end of the study, all scale scores, except for the RSESE, displayed statistically significant Time*Group interactions (**Table 2**). This phenomenon might be partly due to the placebo effect associated with sham stimulation. A review by Thibault found that the placebo effect significantly influences outcomes in neurofeedback training studies mediated by brain electrical signals, a finding that our research also supports (56). We observed statistically significant differences in the RMS values between the T0 and T2 time points in the study group, indicating an enhancement in alpha wave activity compared to baseline following neurofeedback therapy. This finding has been corroborated by another study (22).

The existing research evidence, combined with our study results, offers new insights and theoretical foundations for treating impulsive behavior in male patients with schizophrenia. However, several limitations need to be addressed: 1) Sample Size and Generalizability: The study’s small sample size and single-center design limit the generalizability of the findings. 2) Gender Considerations: The trial exclusively included male patients with schizophrenia, overlooking potential gender differences in impulsive behavior. 3) Assessment Tools: Reliance on a single psychiatric assessment scale may not fully capture the complexity of the clinical symptoms. 4) Focus on Alpha Waves: The neurofeedback intervention and monitoring were limited to alpha waves, without exploring the potential relevance of other brainwave bands to changes in clinical symptoms. These limitations suggest the need for further research with larger, multi-center trials that include both male and female patients and utilize a broader range of neurophysiological markers and psychiatric assessment tools.

## 5 Conclusion

To the best of our knowledge, this is the first clinical study to apply neurofeedback therapy to male schizophrenia patients with impulsive behaviors. Our research demonstrates that six weeks of systematic neurofeedback treatment significantly improves the severity of impulsive behaviors and reduces aggressiveness in these patients. These findings have important implications for decreasing violent incidents and potentially lowering the risk of violent crimes among this population.

## Data Availability

The raw data supporting the conclusions of this article will be made available by the authors, without undue reservation.
